# Lateralization in ^11^C‐Metomidate PET and outcome of adrenalectomy in primary aldosteronism

**DOI:** 10.1002/edm2.368

**Published:** 2022-08-29

**Authors:** Juhani Isojärvi, Marianna Viukari, Ilkka Pörsti, Helena Leijon, Tiina Vesterinen, Marko Seppänen, Pasi I. Nevalainen, Niina Matikainen

**Affiliations:** ^1^ Endocrinology Helsinki University Hospital and University of Helsinki Helsinki Finland; ^2^ Faculty of Medicine and Health Technology Tampere University Tampere Finland; ^3^ Department of Internal Medicine Tampere University Hospital Tampere Finland; ^4^ Department of Pathology University of Helsinki and HUSLAB, Helsinki University Hospital Helsinki Finland; ^5^ Turku PET Centre University of Turku Turku Finland; ^6^ Department of clinical Physiology and Nuclear Medicine Turku University Hospital Turku Finland

**Keywords:** ^11^C‐Metomidate positron emission tomography, adrenal venous sampling, CYP11B2 immunostaining, primary aldosteronism, subtype classification in primary aldosteronism, surgical outcome

## Abstract

**Introduction:**

Subtype classification method is essential when considering adrenalectomy as a possible treatment for primary aldosteronism. We aimed to retrospectively evaluate surgical outcomes of primary aldosteronism in patients who had undergone ^11^C‐metomidate positron emission tomography (^11^C‐MTO‐PET) for subtype classification.

**Methods:**

Postoperative clinical and biochemical cure and histopathological diagnosis from biobank samples were retrospectively evaluated in 44 patients who had all undergone preoperative ^11^C‐MTO‐PET with or without adrenal venous sampling (AVS). We compared those operated based on ^11^C‐MTO‐PET alone and those with concordant or discordant lateralization in ^11^C‐MTO‐PET and AVS studies according to postoperative immunohistochemical findings and biochemical and clinical cure.

**Results:**

Adrenalectomy side was based on ^11^C‐MTO‐PET alone in 14 cases and on AVS in 30 cases of whom 42 achieved complete and two partial biochemical cures. Among those who underwent AVS and were operated according to it, the two lateralization methods were concordant in 22 cases and discordant in 8 cases. Similar immunohistochemical profiles and cure rates were seen after ^11^C‐MTO‐PET alone or AVS‐based operations. Respectively, those with concordant or discordant ^11^C‐MTO‐PET and AVS lateralization did not differ in surgical outcome. Together, we found errors of lateralization diagnostics with ^11^C‐MTO‐PET in 18% and with AVS in 3% among those eligible for adrenal surgery.

**Conclusions:**

Outcomes of adrenalectomy based on clinically significant lateralization in ^11^C‐MTO‐PET alone correspond to those based on ^11^C‐MTO‐PET with concordant AVS lateralization. However, our results suggest that diagnosis of unilateral PA should be performed with caution with ^11^C‐MTO‐PET in case of discordant lateralization studies.

## Graphical abstract

We retrospectively analysed 44 adrenalectomized patients with primary aldosteronism who had all undergone preoperative ^11^C‐MTO‐PET imaging and had adrenal samples available for histopathological diagnosis. Outcomes of adrenalectomy based on clinically significant lateralization in ^11^C‐MTO‐PET alone corresponded to those based on ^11^C‐MTO‐PET with concordant AVS lateralization. However, we found errors with ^11^C‐MTO‐PET method in 18% of lateralization diagnostics among those eligible for adrenal surgery suggesting caution.

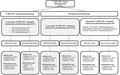

## 1 INTRODUCTION

Primary aldosteronism (PA), the most common cause of secondary hypertension, increases the risk of mortality and cardiovascular and cerebrovascular complications compared to essential hypertension.^1^, ^2^, ^3^, ^4^, ^5^ The risk of adverse outcomes can be reduced by mineralocorticoid receptor antagonists or even more with adrenalectomy in case of lateralization of aldosterone secretion.^6^, ^7^, ^8^, ^9^

Recent meta‐analysis and a prospective cohort study favour surgical treatment over mineralocorticoid receptor antagonists to reduce cardiovascular events and mortality in unilateral PA,^9^, ^10^ but rigorous subtype classification is essential when considering adrenalectomy as a possible treatment. Anatomical imaging with adrenal computed tomography or magnetic resonance imaging has not proven sufficient sensitivity or specificity in most cases but may guide the treatment in young patients with clear PA, hypokalaemia and a solitary nodule.^11^, ^12^, ^13^, ^14^ However, adrenal venous sampling (AVS), the gold standard method for lateralization diagnosis, poses methodological challenges.^11^, ^15^, ^16^ Previous reports suggested that functional imaging with ^11^C‐metomidate positron emission tomography (^11^C‐MTO‐PET) offers an alternative to AVS for PA subtype classification,^17^, ^18^, ^19^ but in our recent prospective study,^20^ ^11^C‐MTO‐PET was inferior to AVS.

In this study, we retrospectively analysed 44 adrenalectomized patients. Patients were eligible if they had undergone preoperative ^11^C‐MTO‐PET imaging, and adrenal biobank samples were available. Our aim was to evaluate in a retrospective cohort the power of ^11^C‐MTO‐PET as a diagnostic method for PA subtype classification according to the surgical outcome of postoperative immunohistochemical analysis and biochemical and clinical cure.

## 2 MATERIALS AND METHODS

### 2.1 Study population

The present analysis is a part of our project, which aims to elucidate the pitfalls of surgical treatment for primary hyperaldosteronism by means of adrenalectomy samples from Finnish biobanks. We retrospectively identified all subjects with a diagnosis of primary hyperaldosteronism (E26.0) who had an adrenalectomy sample available in the Finnish biobanks during 1.1.2000–31.12.2019 and had undergone preoperative ^11^C‐MTO‐PET imaging. Thirty‐four of the subjects fulfilling the inclusion criteria were included in our previous study, which compared lateralization diagnostics between ^11^C‐MTO‐PET imaging and AVS in both operated and medically treated patients.^20^ Patient registry and biobank permissions were obtained, and the study was approved by the Ethics Committee of Helsinki University Hospital. We collected clinical and biochemical data from hospital records.

### 2.2 Lateralization studies

All ^11^C‐MTO‐PET studies were performed in Turku PET Centre without dexamethasone pretreatment, as previously described in detail.^20^, ^21^ All scans were analysed by an expert in nuclear medicine, but the SUVs were inaccessible through biobank data. ACTH‐stimulated AVS was performed at Tampere University Hospital for 36 subjects, 31 of which were successful. The cut points recommended by The Endocrine Society were applied for selectivity and lateralization indexes.^12^ Treatment decisions of subjects were based on clinical judgement in each case.

### 2.3 Histopathological methods

Haematoxylin and eosin‐stained adrenal slides from biobanks were reviewed centrally at the Helsinki University Hospital by a single pathologist with special expertise in adrenal pathology. Immunohistochemical labelling was performed with a previously described primary antibody, CYP11B2 (aldosterone synthase, dilution 1:3000).^22^ Each sample was categorized as aldosterone‐producing adenoma (APA) or non‐APA based on immunohistochemistry as previously described.^20^, ^23^, ^24^

### 2.4 Evaluation of surgical outcome

Biochemical and clinical outcomes for each subject were determined according to the Primary Aldosteronism Surgical Outcome (PASO) criteria.^25^

### 2.5 Statistical analysis

Statistical analysis was performed using IBM SPSS Statistics version 27.0 (IBM Corp., Armonk, NY, USA). Shapiro–Wilk test was applied to test data distribution. Differences between the two subgroups were examined using independent samples t‐test and Mann–Whitney *U*‐test. *p* values <.05 were considered significant.

## 3 RESULTS

**FIGURE 1:**
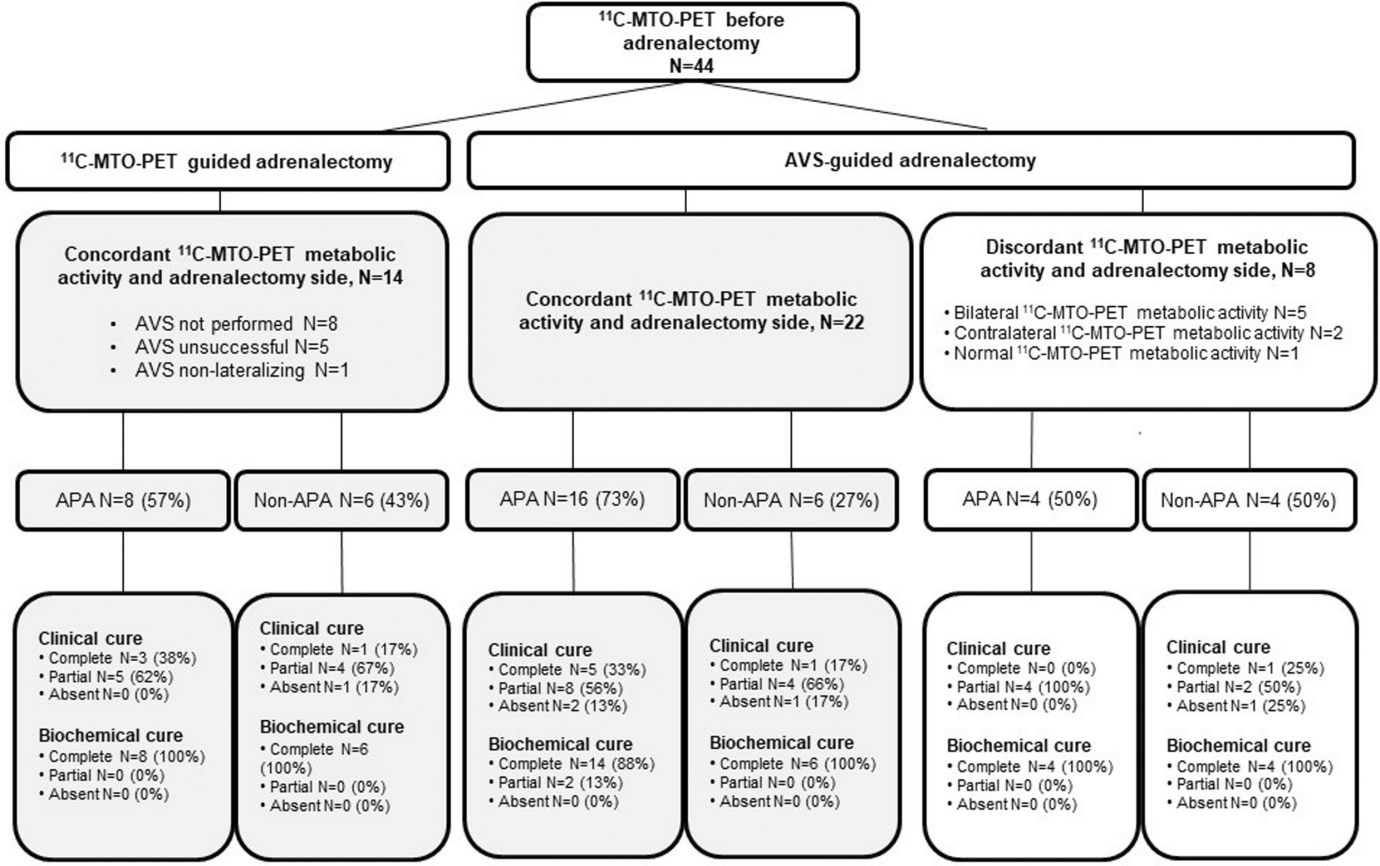
The subjects with an adrenalectomy sample who all underwent ^11^C‐MTO‐PET were divided into those with concordant and discordant ^11^C‐MTO‐PET metabolic activity side with respect to the adrenalectomy side. The concordant group was further divided into AVS‐guided and ^11^C‐MTO‐PET‐guided adrenalectomy groups. The clinical and biochemical outcomes are presented according to the histopathological diagnosis of either APA or non‐APA. ^11^C‐MTO‐PET, ^11^C‐metomidate positron emission tomography; APA, aldosterone‐producing adenoma. Clinical cure was not evaluated for one subject in the concordant group

We identified 44 adrenalectomy specimens in the biobank database from subjects who fulfilled preoperative biochemical criteria for PA^12^ and had undergone preoperative ^11^C‐MTO‐PET imaging with or without AVS (Figure 1). Of the subjects, 14 were operated based on ^11^C‐MTO‐PET alone. The adrenalectomy side was based on AVS lateralization in 30 cases, 22 with concordant and eight with discordant lateralization in ^11^C‐MTO‐PET and AVS studies. Thus, a total of 36 subjects had concordant adrenalectomy and ^11^C‐MTO‐PET metabolic activity side, while eight had discordant ^11^C‐MTO‐PET metabolic activity and adrenalectomy side (Table 1 and Figure 1). Those who were operated based on ^11^C‐MTO‐PET either had or did not have anatomic nodule(s) in adrenal CT. Of these, 5 subjects had an unsuccessful AVS, eight had no preoperative AVS, while one subject showed no detectable lateralization in AVS.

**TABLE 1:** Patient characteristics

	All subjects baseline	All subjects follow‐up	Concordant group baseline	Concordant group follow‐up	Discordant group baseline	Discordant group follow‐up	*p*‐value concordant vs discordant group baseline	*p*‐value concordant vs discordant group follow‐up
Number (male/female)	44 (30/14)	–	36 (24/12)	–	8 (6/2)	–	–	–
Age (years)	56.0 (47.0–61.8)	–	56.0 (48.5–62.0)	–	54.0 (41.0–60.0)	–	0.501	–
BMI (kg/m^2^)	29.5 ± 5.2	–	28.8 ± 5.0	–	32.7 ± 5.1	–	0.050	–
Systolic BP (mmHg)	154 ± 18	133 ± 13	156 ± 19	132 ± 14	148 ± 12	135 ± 9	0.169	0.577
Diastolic BP (mmHg)	93 ± 11	80 ± 9	94 ± 12	80 ± 10	90 ± 6	83 ± 6	0.355	0.222
Duration of treated hypertension (years)	18.0 (13.0–29.0)	–	17.0 (12.0–26.0)	–	20.0 (16.5–30.5)	–	0.211	–
Antihypertensive medication (DDD)	4.0 (2.0–5.3)	2.0 (0.0–3.9)	4.0 (2.0–5.6)	1.5 (0.0–4.0)	4.3 (3.6–4.9)	2.3 (1.3–2.9)	0.780	0.777
Antihypertensive medication (*n*)	2.5 (2.0–3.8)	1.0 (0.0–2.0)	3.0 (1.3–4.0)	1.0 (0.0–2.0)	2.0 (2.0–3.0)	1.0 (1.0–2.8)	0.870	0.777
Plasma aldosterone (pmol/l)	765 (530–1226)	82 (30–208)	806 (543–1414)	87 (30–208)	612 (396–1164)	56 (30–200)	0.314	0.508
PRA (μg/l/h)^a^	0.2 (0.2–0.2)	0.7 (0.2–2.8)	0.2 (0.2–0.2)	0.6 (0.2–3.9)	0.4 (0.2–0.9)	0.9	**0.047**	1.000
ARR, PRA	3360 (1983–5575)	118 (17–503)	3690 (2665–7350)	140 (17–693)	1364 (1001–2451)	82	**<0.001**	0.738
DRC (mU/l)^a^	2.8 (1.7–5.2)	10.2 (5.3–19.5)	2.8 (1.7–5.2)	10.4 (5.6–21.2)	–	7.1	–	0.521
ARR, DRC	338 (93–516)	12 (6–20)	338 (93–516)	13 (6–21)	–	12	–	0.536
Plasma potassium (mmol/l)	2.8 ± 0.4	4.0 ± 0.5	2.9 ± 0.4	4.0 ± 0.5	2.8 ± 0.2	4.0 ± 0.4	0.386	0.932
Plasma sodium (mmol/l)	142.5 ± 2.5	140.3 ± 2.1	142.1 ± 2.4	140.3 ± 2.2	144.4 ± 2.7	140.3 ± 1.6	**0.018**	0.895
Clinical cure complete/partial/absent, *n* (%)	–	11/27/5 (26/63/12)^b^	–	10/21/4 (29/60/11)^b^	–	1/6/1 (13/75/13)	**‐**	0.702
Biochemical cure complete/partial/absent, *n* (%)	–	42/2/0 (95/5/0)	–	34/2/0 (94/6/0)	–	8/0/0 (100/0/0)	–	1.000

*Note*: Data are number, mean ± standard deviation or median (interquartile range). Follow‐up biochemical data were evaluated within a timeframe of a few days to 6 months after adrenalectomy and clinical data at a follow‐up visit after intensification of the medical therapy. The subjects were divided into those with concordant and discordant ^11^C‐MTO‐PET metabolic activity side with respect to the adrenalectomy side.

Abbreviations: ARR, aldosterone‐to‐renin ratio; BMI, body mass index; BP, blood pressure; DDD, defined daily dose; DRC, direct renin concentration; PRA, plasma renin activity. Bold highligts *p*‐values that are considered statistically significant.

^a^ At baseline, renin was measured as PRA in 37 subjects and as DRC in 7 subjects. At follow‐up, renin was measured as PRA in 18 subjects and as DRC in 16 subjects; 10 subjects had no renin measurements at follow‐up, and their biochemical outcome was based on plasma potassium concentrations.

^b^ Clinical cure was not evaluated for one subject in the concordant group.

Two subgroups were determined retrospectively: those with concordant adrenalectomy and ^11^C‐MTO‐PET metabolic activity side (Figure 1, grey background), and those with discordant ^11^C‐MTO‐PET and adrenalectomy side, for example bilateral, contralateral or normal ^11^C‐MTO‐PET metabolic activity (Figure 1, white background and Table 2). We compared these two subgroups according to their biochemical and clinical outcomes following the PASO criteria,^25^ as well as postoperative histopathological diagnosis. Table 1 displays the baseline characteristics for all subjects and subgroups with concordant and discordant PET outcomes. Age did not significantly affect the outcome (data not shown).

**TABLE 2:** Subjects operated based on AVS and with discordant ^11^C‐MTO‐PET metabolic activity side

										Preoperative					Postoperative		
Subject	Age (years)	Sex	Duration of HT (years)	Operation side	Histopathology	Lateralization index	^11^C‐MTO‐PET	Biochemical cure	Clinical cure	P‐Aldo (pmol/l)	PRA (μg/l/h)	ARR	24‐h U‐Aldo (nmol)	P‐K (mmol/l)	P‐Aldo (pmol/l)	P‐K (mmol/l)	Using MRA or K suppl.
**1**	39	M	29	Right	non‐APA	5.9	No lateralization	Complete	Complete	1212	0.9	1347	115	2.9	196	3.8	No
**2**	60	M	18	Right	APA	54.2	No lateralization	Complete	Partial	489	0.8	611	73	2.9	NA	3.8	No
**3**	38	F	15	Left	APA	95.7	No lateralization	Complete	Partial	532	0.2	2660	73	2.4	30	4.1	No
**4**	53	M	31	Left	non‐APA	10.7	No lateralization	Complete	Absent	1020	1.0	1020	71	2.9	210	NA	No
**5**	60	M	16	Right	non‐APA	13.0	No lateralization	Complete	Partial	199	0.2	995	57	2.7	82	3.8	No
**6**	55	F	22	Left	APA	14.2	Contralateral	Complete	Partial	691	0.5	1382	60	2.6	NA	4.4	No
**7**	67	M	36	Right	non‐APA	8.1	Contralateral	Complete	Partial	365	0.2	1825	55	3.0	30	3.5	No
**8**	47	M	18	Right	APA	132.5	Bilateral	Complete	Partial	1230	0.2	6150	57	2.6	30	4.7	No

*Note*: Follow‐up biochemical data were evaluated within a timeframe of a few days to 6 months after adrenalectomy and clinical data at a follow‐up visit after intensification of the medical therapy.

Abbreviations: Aldo, aldosterone; APA, aldosterone‐producing adenoma; ARR, aldosterone‐to‐renin ratio; ^11^C‐MTO‐PET, ^11^C‐metomidate positron emission tomography; F, female; HT, hypertension; M, male; MRA, mineralocorticoid receptor antagonist; NA, not available; PRA, plasma renin activity.

The discordant group had significantly higher plasma renin activity and plasma sodium concentration and lower aldosterone‐renin ratio (ARR) at baseline. Postoperatively, no statistical differences in systolic BP, diastolic BP, use of antihypertensive medication, plasma aldosterone concentration, plasma renin concentration or activity, ARR or plasma potassium concentration were found between these two groups (Table 1).

CYP11B2‐based histopathological diagnosis and the outcome according to the PASO criteria^25^ for clinical and biochemical cure are presented in the Figure 1. Each subject exhibited either APA or non‐APA as the final diagnosis, and the distribution between these histopathological diagnoses did not significantly differ: 67% vs. 33% for concordant and 50% vs. 50% for discordant groups, respectively (*p* = .434). No statistical differences in the cure rates between the groups were detected (Table 1 and Figure 1). One subject was classified as having false negative AVS while after non‐lateralizing AVS he underwent adrenalectomy based on ^11^C‐MTO‐PET lateralization and presented with complete biochemical cure but absent clinical cure. Detailed data for those who underwent AVS‐guided adrenalectomy but showed discordant ^11^C‐MTO‐PET lateralization are shown in Table 2. All these subjects had lateralization index clearly above the cut point of 4 and complete biochemical cure.

## 4 DISCUSSION

The present series is the first report of surgical outcome after adrenalectomy in subjects with discordant metabolic activity side in preoperative functional imaging with ^11^C‐MTO‐PET. Our main findings are as follows: First, those operated based on ^11^C‐MTO‐PET alone or on concordant AVS and ^11^C‐MTO‐PET lateralization were not different regarding adrenalectomy outcome. This suggests that clinically relevant lateralization can be detected in ^11^C‐MTO‐PET. Secondly, AVS‐guided adrenalectomy results in similar cure rates of primary aldosteronism in those with concordant and discordant ^11^C‐MTO‐PET metabolic activity findings. This suggests that diagnosis of unilateral PA should be performed with caution with ^11^C‐MTO‐PET in discordant cases.

In our study, biochemical and clinical cure rates in those with discordant operation and metabolic activity sides were quite good, with 88% demonstrating complete or partial clinical cure, and all subjects demonstrating complete biochemical cure. These results suggest corresponding surgical outcome than among those with concordant operation and metabolic activity side. However, the small number of subjects in the discordant group limits the conclusions. Furthermore, the diagnosis of APA was made in 50% of the cases in the discordant group, indicating that substantial number of the subjects had non‐classic unilateral PA. A recent multicenter study reported increased incidence of cortical hyperplasia in patients without APA who did not achieve cure after adrenalectomy.^26^ Findings of aldosterone‐producing micronodules were also common in our series (data not shown) and may partly explain bilateral or contralateral metabolic activity in ^11^C‐MTO‐PET. However, as shown in Table 2, four cases of APA were found among the eight with discrepancy between AVS and ^11^C‐MTO‐PET but with complete biochemical cure.

Even though the group with ^11^C‐MTO‐PET alone had corresponding outcomes to those with AVS‐guided adrenalectomy, in our whole patient series, reliance on ^11^C‐MTO‐PET would have resulted in unnecessary refraining from surgery in 13.6% of the subjects, and additionally, the non‐culprit adrenal gland would have been operated on in 4.5% of the patients. One subject with non‐lateralizing AVS underwent adrenalectomy based on ^11^C‐MTO‐PET lateralization and achieved complete biochemical cure suggesting false‐negative AVS result even though clinical cure was not achieved. Altogether, this means errors in 18% and 3% of lateralization diagnostics with the ^11^C‐MTO‐PET and AVS methods, respectively, among those eligible for adrenal surgery. In comparison, based on analysis of more than 1300 imaging studies in a recent report, anatomical imaging‐based lateralization would have excluded surgical treatment from 22% of the subjects and resulted in the removal of the non‐culprit adrenal gland from 6% of the subjects.^13^ Previous studies have shown a 36%–39% probability of errors in operation side selection based on adrenal CT, but these numbers included those diagnosed with bilateral disease.^27^, ^28^, ^29^, ^30^

Despite great expectations, the use of ^11^C‐MTO‐PET in the lateralization diagnostics of PA is debated. The present finding that ^11^C‐MTO‐PET does not substitute for AVS in discordant cases with unilateral PA is in line with our prospective study^20^ but disagrees with two others with more favourable conclusions^17^, ^18^ that applied different patient selections and methodology. Indeed, our series also included 14 subjects who were operated based on ^11^C‐MTO‐PET and presented with similar outcomes as the other subjects. Burton et al.^17^ performed PET studies on selected patients based on anatomical findings, and the study by O'Shea et al.^18^ presented a retrospective case series with only one patient undergoing both AVS and ^11^C‐MTO‐PET preoperatively. In a recent prospective study with 20 out of 25 subjects undergoing adrenalectomy, ^11^C‐MTO‐PET performed comparably to AVS and even identified unilateral PA cases missed by AVS.^31^ Besides the small number of subjects included, it must be noted that up to 76% of the study subjects presented with a unilateral adenoma in CT and 90% of adrenalectomized patients showed classic unilateral PA in histopathology. This suggests that ^11^C‐MTO‐PET is indeed useful in case of severe Conn's adenoma and agrees with previous findings by Burton et al. who aimed to recruit patients with a unilateral aldosteronoma.^17^ However, the ability of ^11^C‐MTO‐PET to diagnose unilateral non‐classic forms of PA that are becoming more prevalent remains unanswered by these studies. Altogether, differences in patient selection may partly explain the discrepancies between our results and those by Puar et al.^31^

A limitation of the current study is that contrary to the protocol developed by Burton et al.,^17^ our PET protocol did not include pretreatment with dexamethasone. Therefore, background CYP11B1 activity, that is tracer uptake by cortisol synthase, may decrease the SUV differences in the PET scan. Although CYP11B1 activity is suppressed by dexamethasone pretreatment, the effect of dexamethasone on CYP11B2 activity, ^11^C‐MTO uptake, and lateralization of aldosterone secretion through inhibition of ACTH secretion remains unknown. However, more than 50% suppression of plasma aldosterone concentration has been demonstrated after dexamethasone pretreatment in both APA and bilateral hyperplasia.^21^, ^32^, ^33^ The main problem with the ^11^C‐MTO isotope tracer is its selectivity for both CYP11B1 and CYP11B2, which is an obstacle for non‐invasive subtype diagnostics in PA, and more specific tracers are awaited.^34^

The strength of the current approach is ascertainment of the diagnosis by including only those undergoing surgical treatment and by using immunohistochemistry in addition to assessing postoperative cure. However, further studies are needed to address the best method to exclude false positive lateralization in medically treated bilateral aldosteronism. Also, false‐negative lateralization leading to inadvertent medical treatment and lack of adrenalectomy cannot be evaluated in this setting. The size of the study population and retrospective approach are limitations of our study. In addition, the postoperative evaluation time point deviated from the 6‐month time defined in the PASO criteria^25^ in some of the subjects.

In summary, our real‐world results suggest that AVS and ^11^C‐MTO‐PET‐guided lateralization diagnostics lead to similar adrenalectomy outcomes but suggest caution when lateralization in AVS and ^11^C‐MTO‐PET is discordant, which poses a risk of subtype misclassification with ^11^C‐MTO‐PET.

## AUTHOR CONTRIBUTIONS

**Juhani Isojärvi:** Formal analysis (lead); writing – original draft (equal). **Marianna Viukari:** Data curation (lead); formal analysis (supporting); writing – original draft (equal); writing – review and editing (equal). **Ilkka Pörsti:** Conceptualization (supporting); writing – review and editing (equal). **Helena Leijon:** Data curation (equal); methodology (equal); writing – review and editing (equal). **Tiina Vesterinen:** Data curation (equal); methodology (equal); writing – review and editing (equal). **Marko Seppänen:** Methodology (equal); writing – review and editing (equal). **Pasi I Nevalainen:** Conceptualization (supporting); writing – review and editing (equal). **Niina Matikainen:** Conceptualization (lead); funding acquisition (lead); methodology (lead); project administration (equal); writing – review and editing (lead).

## FUNDING INFORMATION

This work was supported by a research grant from Helsinki University Hospital (VTR TYH2018111 and TYH2020402) to N.M.

## CONFLICT OF INTEREST

The authors declare that the research was conducted in the absence of any commercial or financial relationships that could be construed as a potential conflict of interest.

## ETHICAL APPROVAL

This study was performed in line with the principles of the Declaration of Helsinki. Approval was granted by the Ethics Committee of University Helsinki (7.11.2018/ HUS/1352/2018.)

## Data Availability

The data that support the findings of this study are available from the corresponding author upon reasonable request.
